# Recent advances in dynamic m^6^A RNA modification

**DOI:** 10.1098/rsob.160003

**Published:** 2016-04-13

**Authors:** Guangchao Cao, Hua-Bing Li, Zhinan Yin, Richard A. Flavell

**Affiliations:** 1 State Key Laboratory of Medicinal Chemical Biology, College of Life Sciences, Nankai University, Tianjin 300071, People's Republic of China; 2 Department of Immunobiology, School of Medicine, Yale University, New Haven, CT 06520, USA; 3 The First Affiliated Hospital, Biomedical Translational Research Institute, Guangdong Province Key Laboratory of Molecular Immunology and Antibody Engineering, Jinan University, Guangzhou 510632, People's Republic of China; 4 Howard Hughes Medical Institute, Chevy Chase, MD 20815, USA

**Keywords:** m^6^A, mRNA, methylation

## Abstract

The identification of m^6^A demethylases and high-throughput sequencing analysis of methylated transcriptome corroborated m^6^A RNA epigenetic modification as a dynamic regulation process, and reignited its investigation in the past few years. Many basic concepts of cytogenetics have been revolutionized by the growing understanding of the fundamental role of m^6^A in RNA splicing, degradation and translation. In this review, we summarize typical features of methylated transcriptome in mammals, and highlight the ‘writers’, ‘erasers’ and ‘readers’ of m^6^A RNA modification. Moreover, we emphasize recent advances of biological functions of m^6^A and conceive the possible roles of m^6^A in the regulation of immune response and related diseases.

## 1. Introduction

RNA serves as an inevitable connecting link for genetic information passing from DNA to protein. The intimate relationship between mRNA and protein makes it accredited to present mRNA data for gene expression when protein levels are difficult to address. However, cellular protein levels are not necessarily correlated with mRNA levels [1,2], suggesting that post-transcriptional mRNA regulation plays an important role in gene expression. Indeed, more than 100 types of chemical modification have been identified in cellular RNA (including rRNA, tRNA, snRNA, mRNA and long-non-coding RNA) in recent decades [3,4], among the most prevalent internal mRNA/lncRNA modifications of which is *N*^6^-methyladenosine (m^6^A). Discovered in the 1970s [5–8], m^6^A has been observed in a wide range of eukaryotes, ranging from yeast, *Arabidopsis thaliana*, *Drosophila* to mammals, and is also found in the RNA of viruses [9–11]. However, owing to a lack of knowledge of m^6^A demethylating enzymes and the short life of most RNA species (median mammalian RNA half-lives are approx. 5 h [12,13]), m^6^A modifications had long been considered to be static and unalterable. The inability to identify m^6^A-containing mRNAs also hindered investigation of the biological roles of this chemical modification.

In 2011, the discovery of fat mass and obesity associated protein (FTO) as the first genuine m^6^A demethylase revived interest in mRNA/lncRNA methylation [14], because it defined m^6^A RNA modification as a dynamic process and its disturbance probably correlated to human diseases. Two independent studies developed an m^6^A RNA immunoprecipitation approach followed by high-throughput sequencing (MeRIP-seq) in 2012 that defined the methylated transcriptome in mammals [15,16]. These results demonstrated for the first time that m^6^A was a prevalent mRNA modification, and reignited the investigation on m^6^A ‘writers’, ‘erasers’, ‘readers’ and their physiology functions. Recent studies have already shown that dysregulation of this modification may contributes to obesity, brain development abnormalities and other diseases [17–22], thus emphasizing the importance of m^6^A RNA modification.

In this review, we discuss recent studies that profiled the features of methylated transcriptome. We also highlight the dynamic regulation of m^6^A RNA modification by adenosine methyltransferases (‘writers’) and demethylases (‘erasers’). Furthermore, we emphasize recent advances on the role of m^6^A RNA modification in biological processes and human diseases. Finally, we give some perspective for further investigation and conceive the possible role of m^6^A in the regulation of innate and adaptive immunity.

## 2. Widespread m^6^A mRNA/lncRNA modification

In the 1970s, several groups found that polyadenylated RNA from mammalian cells contained the most abundant chemical modification, m^6^A [5–8]. However, poly(A) RNA could have arisen from mitochondrial RNA, tRNA and rRNA, because these RNAs contains poly (A) tracts [23–25]. Besides, very few defined internal mRNAs were shown to contain m^6^A, which led to doubts whether m^6^A was indeed a prevalent modification in mRNA and whether this modification played any important role in biological processes. In 2012, two studies rested these doubts using MeRIP-seq techniques showing that thousands of mRNAs and lncRNAs contained m^6^A [15,16], unequivocally demonstrating that m^6^A is a widespread modification in mRNA.

These two studies and results from other groups published recently presented a striking finding that m^6^A residues were enriched in 5′ untranslated regions (UTRs), around stop codons and in 3′ UTRs adjacent to stop codons in mammalian mRNAs [15,16,26,27]; in *Arabidopsis thaliana*, m^6^A is also enriched around the start codons [28]. Many mRNA binding proteins bind to the 3′ UTR [29], which is the most structured portion of mRNAs [30,31]. m^6^A was reported to marginally reduce the stability of A : U base pairing [32]; methylated transcripts in meiotic yeast were less structured [33]; and a recent study found that m^6^A-dependent RNA structural switches regulated RNA–protein interactions to affect the abundance as well as alternative splicing of target mRNAs (see below) [34]. These results indicated that the methylation regions' specificity of m^6^A was intimately correlated with their unique regulatory functions.

The question is how this region-specific methylation is targeted. Bioinformatic analysis of MeRIP-Seq data using the motif discovery algorithm finding informative regulatory elements (FIRE) identified the predominant consensus motifs of m^6^A: G [G/A] m^6^ACU and related variants ([AC]GAC[GU], GGAC, [AU][CG]G[AG]AC and UGAC), and almost 90% of all m^6^A peaks contain at least one of the motifs [16]. This consensus motif is extremely similar to the identified sequence obtained from mutational studies and substrate preference of methyltransferase enzyme *in vitro* in the 1970s: [G/A/U] [G/A] m^6^AC [U/A/C] [35–39]. Other methylation motifs were also identified, but were much less prominent [15,16]. These data suggest that the adenosine methyltransferases and demethylases may also constitute a limited repertoire with predominant and a few less abundant elements.

Another interesting phenomenon was that only a minor part of mRNA transcripts were m^6^A modified. This was not due to the lack of consensus motifs in some mRNAs, as GAC motif is commonly found approximately every 64 nucleotides in RNA. In fact, the majority of m^6^A consensus motifs were not methylated, and more importantly, there may be only some copies of an mRNA transcript that were modified [15,16,18,26,27]. These results potentiate the concept that m^6^A mRNA modification as a dynamic process. However, some undefined sequences around these consensus motifs that could regulate the methylation status may exist, and specific structures of certain mRNAs may also explain the relatively low percentage of m^6^A-modified mRNAs. Thus, the development of techniques to map the m^6^A sites at single-nucleotide resolution would help to address these questions. IP-based cross-linking-assisted approaches were developed by several groups for the mapping of mammalian mRNAs [40–42], and high-resolution mapping of yeast m^6^A was also achieved [33].

## 3. m^6^a writers—adenosine methyltransferases

### 3.1. METTL3

A multiprotein methyltransferase complex was shown to mediate m^6^A mRNA methylation [43–45], and METTL3 was earlier identified as a *S*-adenosyl-l-methionine (SAM)-binding component of this complex [45] and could exhibit catalytic functions by itself [46]. Knockdown METTL3 reduced m^6^A peaks in mRNAs from mouse embryonic stem cells, Hela cells and HepG2 cells [15,26,27,47]. These results defined METTL3 as a methyltransferase for m^6^A RNA modification. Genetic ablation of METTL3 in blastocysts generated by mating of METTL3^+/−^ mice led to almost complete depletion of m^6^A on mRNAs, further emphasizing the critical role of METTL3 in m^6^A modification [18]. Also, METTL3 is highly conserved in eukaryotes, and homologues in yeast, plant and *Drosophila* have also been identified [48–50]. Both nuclear and cytoplasmic localization of METTL3 were observed [27,33], suggesting that mRNA methylation could occur in both nucleus and cytoplasm, which is consistent with early studies showing that cytosolic extracts also possessed methyltransferase activity [51].

### 3.2. METTL14

METTL14 was a close homologue of METTL3 [52]. Purified METTL14 could also specifically methylate the consensus GAC motifs by itself [46,47], and knockdown of METTL14 could also lead to decreases of m^6^A content in mRNAs. Further studies revealed that these two components form a complex in cells and the methylation activity of this complex was much more efficient than separated parts [46,47].

### 3.3. WTAP

Wilms tumour 1-associated protein (WTAP) was known to be involved in mRNA splicing [53]. The important role of WTAP in m^6^A methylation was first established in yeast and *Arabidopsis thaliana* by studying its homologues Mum2 and FIP37, respectively, which were found to associate with METTL3 and were required for efficient methylation of mRNA [48,54]. Recent researches revealed that mammalian WTAP also interacts with the METTL3–METTL14 core complex [46,55]. Although WTAP alone did not show any methyltransferase activity *in vitro*, knockdown of WTAP strikingly reduced the m^6^A peaks in cellular mRNAs, even more significantly than knockdown of METTL3 or METTL14 [46]. Further research uncovered that one of the possible mechanisms of regulating methylation by WTAP was to facilitate METTL3–METTL14 translocation to nuclear speckles [55]. Furthermore, a recent study identified WTAP-dependent and -independent m^6^A modification sites characters in multiple dynamic systems. WTAP-dependent sites were located at internal positions and topologically static, whereas WTAP-independent sites contributed as part of the cap structure at the transcription start bases [56]. These results suggested different regulation patterns of mA6 methylation in a region-dependent way.

### 3.4. KIAA1429

As mentioned previously, the m^6^A methyltransferase complex is a multicomponent extract, suggesting other candidates may be involved in this process. A recent study revealed 13 candidates associating with known methyltransferase components by a proteomic approach. siRNA depletion experiments revealed that one of the candidates, KIAA1429, was required for the full methylation programme in mammals [56].

## 4. m^6^a erasers—demethylases

### 4.1. FTO

The discovery of FTO as the first m^6^A mRNA demethylase entrenched the conception of m^6^A as reversible modification [14]. This study found that both DNA and RNA were substrates of FTO-mediated demethylation, and knockdown of FTO increased m^6^A peaks while over expression experiments reduced them [14]. Later investigations revealed the oxidation of m^6^A by FTO and discovered two new intermediate modifications, 6-hydroxymethyladenosine and 6-formyladenosine [57]. The correlation of FTO dysregulation with obesity, brain malformations and growth retardation was also reported, and suggested m^6^A may have important regulatory functions in these diseases [22,58–60].

### 4.2. ALKBH5

FTO is a member of the ALKB family. Another member of this family, ALKBH5, was also identified as a demethylase, as knockdown of this protein in human cell lines yielded higher m^6^A mRNA peaks [61]. ALKBH5 catalytic reaction directly removes the methyl group from m^6^A-methylated adenosine instead of oxidative demethylation, which was different from FTO [61]. *Alkbh5*-knockout mice showed a marked increase of apoptotic cells in the testes, indicating a defect in spermatogenesis [61]. Later studies found that, in addition to mRNAs, other types of nuclear RNAs were also substrates of ALBKH5 [62].

## 5. m^6^a readers—binding proteins

Each component of an organism, eukaryotic or prokaryotic, coordinates with each other to construct a concerted system, and modulating these components would lead to subsequent biological consequences. It is conceivable that m^6^A mRNA modification performs its function through two main approaches: fine-tuning the structure of the methylated transcripts to block or induce protein–RNA interactions, or being directly recognized by m^6^A binding proteins to induce subsequent reactions.

### 5.1. HNRNPC and HNRNPA2B1

Biochemical approaches have verified the structural alternations in m^6^A-modified RNAs, favouring the transition from paired to unpaired RNA [63]. Recent publications uncovered that m^6^A destabilized the stacking properties of the region around its opposing U-tracts in the hairpin-stem of RNA transcripts, which made the U-tracts more single-stranded or accessible, thus enhancing its binding with heterogeneous nuclear ribonucleoprotein C (HNRNPC) [34,64]. HNRNPC is an abundant nuclear RNA binding protein known to be involved in pre-mRNA processing [65–69], and further research revealed that the modulation of HNRNPC–RNA binding by m^6^A affected the abundance and alternative splicing of target transcripts [34]. Another example of this structure alternation mediating regulating protein–RNA interaction was HuR; the m^6^A modification affected its ability to bind to different RNA probes *in vitro* [47].

FIRE analysis revealed highly significant enrichment of the RGAC element among the binding sites of another member of the HNRNP family, HNRNPA2B1, suggesting HNRNPA2B1 as an m^6^A reader candidate. Further research showed that HNRNPA2B1 cross-linking-induced deletions performed 20-fold higher overlaps with m^6^A peaks compared with background deletions, strongly supporting HNRNPA2B1 as an m^6^A reader by directly binding to a subset of m^6^A consensus sequences. Furthermore, HNRNPA2B1 interacts with the DGCR8 protein, a component of the pri-miRNA microprocessor complex, and facilitates the processing of pri-miRNAs [70].

### 5.2. YTHDF2 and YTHDF1

Several mammalian proteins were identified as selective m^6^A binding proteins. YTHDF1–3 were reported to possess much higher binding affinity to methylated probe compared with the unmethylated one [15,71]. All of these three members of YTH domain family showed preferential binding with m^6^A-containing mRNAs *in vitro*. Knockdown experiments suggested that YTHDF2 binding affected the cognate mRNA degradation process as these mRNA targets showed decreased half-lives. Further investigation found that binding with YTHDF2 resulted in mRNA localization to mRNA decay sites such as processing bodies (P-bodies) for accelerated degradation [71]. Another report showed that YTHDF2 preserves 5′UTR methylation of stress-induced transcripts by limiting the m^6^A ‘eraser’ FTO from demethylation, and the increased 5′UTR methylation in the form of m^6^A promotes cap-independent translation initiation [72]. These results suggested diverse roles of YTHDF2 under different circumstances.

Recently, *in vivo* binding of m^6^A by YTHDF1 was also demonstrated. Knockdown of YTHDF1 reduced ribosome occupancy and decreased translation of m^6^A-modified mRNAs. Further investigation revealed that YTHDF1 interacts with initiation factors to promote translation [73]. These results presented a novel mechanism of translation regulation by m^6^A modification in mRNA.

### 5.3. eIF3

Most recently, Meyer *et al.* [74] reported that eukaryotic initiation factor 3 (eIF3), a component of 43S translation preinitiation complex, directly binds with 5′ UTR m^6^A. Cross-linking of eIF3 to the m^6^A-containing RNA probe was substantially increased compared with the A-containing probe, and eIF3 preferably bind to Gm6AC nucleotides. Further research revealed that eIF3-binding sites were primarily localized to 5′ UTRs of mRNAs, which was important in regulating translation initiation. Restraining m^6^A modification by FTO overexpression substantially depleted mRNAs that contain a high stoichiometry m^6^A site within their 5′ UTR in the eIF3-bound fraction, indicating that eIF3 interacts with mRNAs in an m^6^A-dependent manner in cells. Moreover, the researchers found that the binding of eIF3 to 5′ UTR m^6^A was independent of YTHDF1, which was reported to interact with eIF3, thus supporting the idea that eIF3 was able to directly bind m^6^A [74].

## 6. m^6^A and miRNAs

m^6^A mRNA modification were enriched in 5′ UTRs, around stop codon and in the proximal region of 3′ UTRs, whereas miRNAs-targeted sites at the 5′ end and 3′ end of 3′UTRs suggested a potential link between m^6^A modification and miRNA targeting sites [15,16,26,27]. A recent study also showed that m^6^A peaks were enriched at miRNAs target sites [27]. Further research verified this hypothesis using *Dicer* knockdown and overexpression approaches, which showed that m^6^A abundance was positively correlated with Dicer level, which mediates miRNA maturation. ASF (a nuclear speckle marker) staining revealed that knockdown of Dicer, which presents in both the nucleus and cytoplasm [75], resulted in disrupted localization of METTL3 to nuclear speckles without affecting METTL3 abundance. Consistently, experiments by knockdown, overexpression and mutation of certain miRNAs showed that miRNAs regulated the m^6^A methyltransferase activity of METTL3 by modulating its binding to mRNAs in a sequence-dependent manner [27].

More recently, m^6^A modification was also identified on primary microRNAs (pri-miRNAs). Researchers found that METTL3 methylates pri-miRNAs, marking them for recognition and processing by DGCR8. METTL3 depletion reduced the binding of DGCR8 to pri-miRNAs, and resulted in the global reduction of mature miRNAs and concomitant accumulation of unprocessed pri-miRNAs [76]. Moreover, the ‘reader’ that recognized the m^6^A modification on pri-miRNAs (i.e. HNRNPA2B1) was also identified. HNRNPA2B1 interacts with DGCR8 to promote pri-miRNA processing [70]. These results revealed tight regulations among m^6^A modification, miRNA biogenesis and function.

## 7. m^6^a function—effects on mRNA fate and biological consequences

During the past 3–4 years, the breakthrough of developing transcriptome-wide profiling of m^6^A led to the feature of crucial regulatory roles of m^6^A modification in a wide range of fundamental cellular processes, including gene expression, meiosis, stemness and circadian rhythm. The writers, erasers and readers were also found to have intimate relevance to certain diseases, such as obesity, infertility and growth retardation. Considering the prevalent distribution of m^6^A modification in mRNAs and lncRNAs, it would not be surprising to uncover more specific regulatory roles of m^6^A along with identification of more m^6^A readers.

### 7.1. Effects on mRNA fate—splicing, degradation and translation

The localization of METTL3, METTL14, WTAP and ALKBH5 were mainly found in nuclear speckles, and FTO was also found partially co-localized with nuclear speckles, a well-known site for pre-mRNA processing [77–81]. These phenomena raised the prospect of a regulatory role of m^6^A in mRNA splicing. Knockdown of WTAP or METTL3 indeed generated different mRNA isoforms, and WTAP was a known splicing factor [53,55]. Also, ALKBH5 was shown to affect the rate of splicing [61]. All of these results supported the hypothesis that m^6^A may be involved in mRNA splicing. A recent study demonstrated that m^6^A-mediated mRNA structure remodelling affected binding to HNRNPC, which was an abundant nuclear RNA binding protein responsible for pre-mRNA processing, and alternative splicing [34]. Indeed, knockdown of Mettl3/14 co-regulated the expression of 5251 genes with HNRNPC knockdown in HEK293T cells, and 890 of these genes were in high confidence in containing m^6^A-mediated structure remodelling switch. Further research indicated that this remodelling tended to regulate splicing events at nearby exons. The regulatory role of m^6^A in mRNA splicing was also reported in the study of FTO-depleted 3T3-L1 pre-adipocytes. The researchers found that enhanced m^6^A level in response to FTO depletion promotes RNA binding ability of splicing regulatory protein SRSF2, leading to increased inclusion of target exons [17]. These data provide strong evidence on a mechanistic relationship between the presence of m^6^A and splicing events.

**Figure 1.**
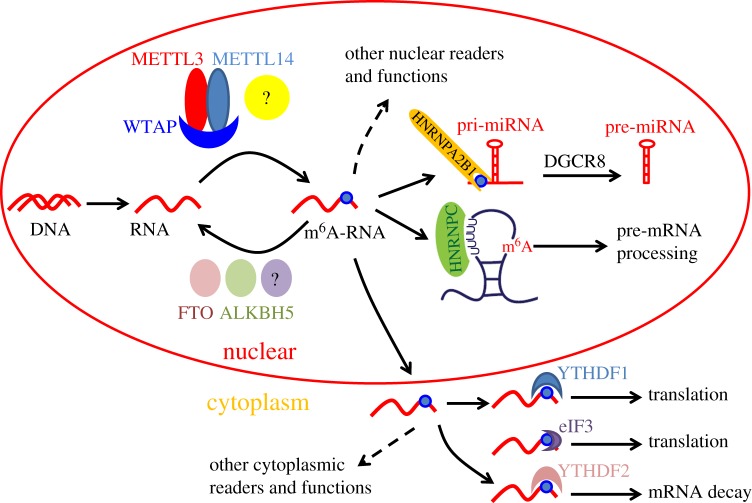
Dynamic m^6^A RNA modifications and mediated functions. m^6^A mRNA methylation is mediated by a multiprotein complex that includes METTL3, METTL14 and WTAP, whereas demethylases, such as FTO and ALKBH5, erase m^6^A. Recognition of m^6^A by HNRNPC in the nucleus mediates alternative splicing of pre-mRNA, and HNRNPA2B1 promotes pri-miRNA processing to pre-miRNA. In cytoplasm, binding of m^6^A sites with different readers mediates divergent functions. YTHDF1 binds m^6^A-modified mRNAs through interactions with initiation factors and ribosomes to increase translational output, and eIF3 can also directly bind to 5′UTR m^6^A to initiate translation, whereas m^6^A recognition by YTHDF2 leads to mRNA decay. More nuclear and cytoplasmic readers need to be defined to illuminate the functions of m^6^A in mRNA export, translation and storage.

Cellular mRNAs possess fast turnover with a median half-life of about 5 h. The dynamic mRNA synthesis and degradation render cells liable to make rapid adjustment in response to environmental changes via newly degraded nucleotides for de novo synthesis. Thus, the identification and regulation of certain mRNAs for degradation is vitally important. Knockdown of METTL3 or METTL14 in mouse embryonic stem cells modestly increased the stability of target mRNAs, suggesting that m^6^A modification induces mRNA instability [18,47]. Further studies revealed that the binding activities of HuR, a known mRNA stabilizer, were impaired by m^6^A modification adjacent to the binding sites *in vitro* [47]. The regulatory role of m^6^A on mRNA degradation was verified by the discovery that binding with YTHDF2 promoted thousands of cellular mRNA degradation via translocation to decay sites [71] (figure 1). Another m^6^A reader—HNRNPC—may also regulate mRNA degradation, because knockdown of HNRNPC also affected the abundance of target transcripts [34]. Knockdown of an m^6^A eraser—ALKBH5—increased poly(A) mRNAs in the nucleus [61], suggesting that ALKBH5 and its demethylation activity may affect mRNA export from nucleus to cytoplasm, or nascent mRNA synthesis.

Organisms perform their biological functions mainly through proteins. Because mRNAs are the direct templates for protein synthesis, the enrichment of m^6^A in exons and around the stop codon regions makes it conceivable that m^6^A may also regulate translation. In a recent study performed in mouse embryonic stem cells (mESCs) and embryoid bodies (EBs), METTL3 ablation modestly yet significantly increased translation efficiency, indicating a regulatory role of m^6^A in translation [18]. More recently, another m^6^A reader, YTHDF1, was reported to interact with initiation factors and ribosomes to increase translational output [73], presenting direct evidence for translational regulation functions of m^6^A. One of the translation initiation factors, eIF3, was also reported to directly bind 5′ UTR m^6^A, which was sufficient to recruit the 43S complex to initiate translation in the absence of the cap-binding factor eIF4E [74] (figure 1). Furthermore, the researchers also found that diverse cellular stresses induced a transcriptome-wide redistribution of m^6^A, resulting in increased numbers of mRNAs with 5′ UTR m^6^A, which thus presented a concept of dynamic m^6^A events in response to stress. The identification of more m^6^A readers will help to better elucidate the translation process.

### 7.2. Biological consequences of m^6^A—dysregulation in cellular processes and diseases

#### 7.2.1. Stemness—mammalian embryonic stem cell fate transition

Several groups have reported the prevalent m^6^A mRNA modification in mammalian embryonic stem cells and a similar region distribution with somatic cells [18,26,27,47]. However, the regulatory role of m^6^A modification in cell fate transition in ESCs was conflictive among these studies. The earliest reports showed that knockdown of METTL3 or METTL14 via shRNA interfering led to decreased proliferation rate of mESCs; RT-qPCR of pluripotency factors displayed reduction in knockdown cells, whereas developmental regulators were increased. Enrichment of developmental factors rather than pluripotency-related genes were also found in METTL3 and METTL14 targets, and m^6^A methylation destabilized these transcripts via damping the binding with HuR. Based on these observations, a logical deduction was made that m^6^A methylation was required to keep mESCs at ground state [47]. Another group overexpressed METTL3 in mouse embryonic fibroblasts (MEFs) and found a significant increase in m^6^A abundance, enhanced expression of key pluripotent factors and improvement of reprogramming efficiency. Reverse results were also found in METTL3 knockdown and methionine adenosyltransferase inhibitor-treated cells, indicating that m^6^A was required for MEF reprogramming to pluripotency [27].

Contrary to these results, genetic knockout or shRNA knockdown of *Mettl3* demonstrated improved self-renewal in mESCs reported in a recent study [26]. The same group also found impaired differentiation towards cardiomyocytes and the neural lineage *in vitro* in *Mettl3* KO mESCs which retained high levels of pluripotency regulator Nanog expression. The *in vivo* teratoma generation experiments also showed poorly differentiated cells in teratomas derived from KO ESCs with higher staining of NANOG and the proliferation marker KI67 [26]. These results suggested that m^6^A suppresses self-renewal and promotes differentiation.

The conflict was rested with the generation of Mettl3 KO ESCs by mating of Mettl3^+/−^ mice. Mettl3 KO embryonic blastocysts failed to adequately repress pluripotent genes, and differentiated into mature neurons *in vitro* and poorly differentiated in teratomas *in vivo*, and also hampered priming from naive pluripotent state towards an epiblast-like state [18]. These results indicated that depletion of m^6^A modification blocked differentiation in ESCs and led to a hypernaive pluripotenct state. Further research adopted an siRNA interfering approach to knockdown METTL3 in mESCs in both naive pluripotent state and primed EpiSC state. Quantitative PCR results showed upregulation of both pluripotent regulators and developmental factors upon knockdown of METTL3 in mESCs in either state. However, the basal transcript levels of pluripotency genes are abundant, whereas lineage factors are extremely low under naive state. When progressing towards the primed state, the pluripotency genes were downregulated and lineage commitment makers became abundantly expressed. Thus, the obliteration of METTL3 potentiated the already high pluripotency genes in the naive condition to create a hypernaive pluripotent state, but mounted the dominating developmental factors in the primed state and tipped the balance towards differentiation [18]. Most recently, Aguillo *et al.* [82] showed that ZFP217 sequesters METTL3 and diminishes METTL3 binding with RNAs to restrain m^6^A modification, and that low m^6^A levels in ESC-related transcripts enable pluripotency and reprogramming.

These results demonstrated that m^6^A modification determined the fate transition in mESCs. Also, m^6^A methylomes in human and mouse ESCs were shown to be highly conserved [26], and a recent research showed an important role of METTL3 homologue in the development of *Arabidopsis* embryo [83], which suggested a conserved role of m^6^A in ESC development.

#### 7.2.2. Obesity—FTO in adipogenesis

Genome-wide association studies linked common variants of FTO gene with childhood and adult obesity in 2007 [58–60]. Loss-of-function mutation in the FTO gene is responsible for a recessive lethal syndrome, including postnatal growth retardation, microcephaly and cardiac defects [22]. Studies by inactivation or overexpression of FTO in mice suggested that FTO tended to promote obesity and metabolic syndrome by driving obesity-prone behaviours such as increased food intake [84–87], consistent with its highest expression level in the brain [88]. The finding that FTO-mediated m^6^A demethylation controls exonic splicing of adipogenic regulatory factor RUNX1T1 emphasized the regulatory role of FTO in adipogenesis [17]. Another group reported that obesity variants within the *FTO* gene formed a long-range connection with IRX3 [89], which was located downstream from *FTO*, and deficiency in this gene resulted in 25–30% body weight loss [90], thus questioning a direct role for FTO in obesity.

m^6^A mRNA modification has also been shown to exert regulatory functions in apoptosis, circadian rhythm and meiosis, and aberrant m^6^A mRNA modifications are correlated to a variety of human diseases, including cancer, infertility and hepatitis, which has been reviewed elsewhere [19–21,91,92].

## 8. Possible role of m^6^A in immune response

The immune system serves as the security guard of the human body, and plays a most important role in clearance of pathogens, either endogenous or exogenous. The dysfunction of the immune system is involved in almost all known human diseases, including cancers, infection diseases, inflammation diseases, allergies, metabolism syndromes and autoimmune diseases. The immune system is composed of two parts—the innate and adaptive immune system. The innate immune reactions are rapid and non-specific, whereas the adaptive response needs antigen presentation, clone expansion and differentiation to perform antigen-specific reactions [93,94]. The abundance of antigens in the environment and the quick turnover of apoptotic internal cells demands rapid adjustment abilities of immune cells. Indeed, upon antigen recognition, innate immune cells and memorial adaptive cells are capable of releasing a robust amount of cytokines in as little as 2 h [95], which is called ‘cytokine storm’ and is unlikely to be driven from de novo gene transcription. As discussed above, the fast turnover of mRNAs is an energy-cost-effective process in responding to environmental changes compared with proteins. Because m^6^A plays critical roles in mRNA splicing, degradation and translation, it is conceivable that it may also play an important role in immune reactions. In fact, m^6^A has been shown to protect RNA from recognition by TLR3 and TLR7 as invasive species for degradation [96,97]. Also, one of the erasers of m^6^A, ALKBH5, has been shown to be highly expressed in the spleen and lung, organs enriched in immune cells and with frequent immune reactions [61]. Performing experimental immune disease models using *FTO*, *Alkbh5* and *Mettl3* knockout mice may help elucidate the role of m^6^A in immune response.
